# The Role of Osteokines in Sarcopenia: Therapeutic Directions and Application Prospects

**DOI:** 10.3389/fcell.2021.735374

**Published:** 2021-09-28

**Authors:** Wenhao Lu, Wenfeng Xiao, Wenqing Xie, Xin Fu, Linyuan Pan, Hongfu Jin, Yongle Yu, Yi Zhang, Yusheng Li

**Affiliations:** ^1^Department of Orthopaedics, Xiangya Hospital, Central South University, Changsha, China; ^2^National Clinical Research Center for Geriatric Disorders, Xiangya Hospital, Central South University, Changsha, China; ^3^Xiangya School of Medicine, Central South University, Changsha, China

**Keywords:** osteokines, FGF-23, IGF-1, RANKL, osteocalcin, sarcopenia

## Abstract

Sarcopenia is an age-related disease in which muscle mass, strength and function may decline with age or can be secondary to cachexia or malnutrition and can lead to weakness, falls and even death. With the increase in life expectancy, sarcopenia has become a major threat to the health of the elderly. Currently, our understanding of bone-muscle interactions is not limited to their mechanical coupling. Bone and muscle have been identified as secretory endocrine organs, and their interaction may affect the function of each. Both muscle-derived factors and osteokines can play a role in regulating muscle and bone metabolism via autocrine, paracrine and endocrine mechanisms. Herein, we comprehensively summarize the latest research progress on the effects of the osteokines FGF-23, IGF-1, RANKL and osteocalcin on muscle to explore whether these cytokines can be utilized to treat and prevent sarcopenia.

## Introduction

The elderly population worldwide is increasing rapidly and aging at a high rate. At the same time, aging-related diseases are also on the rise. The global proportion of the population over 60 years old was 10% in 2000 and is expected to reach 21.8% by 2050 and 32.2% in 2100 (Lutz et al., 2008; Kanasi et al., 2016). This means that the world will become an aging society, which will not only increase burdens on the working-age population but also lead to transformation of the disease spectrum: in the past, infectious diseases and nutritional deficiencies were the primary global health issues, but soon, degenerative diseases, diabetes, cardiovascular disease, chronic respiratory diseases and cancer, which are chronic non-communicable diseases, will become the primary focus (Abegunde et al., 2007). The aging process can cause significant changes in many tissues and organs, especially skeletal muscle (Lauretani et al., 2003). For 40- to 80-year-old people, the total skeletal muscle mass was found to be reduced by 30 to 50%, regardless of sex (Deschenes, 2004; Faulkner et al., 2007). In 1988, Rosenberg noted that muscle mass gradually decreases with age and defined this disease as sarcopenia (Rosenberg, 1997). A meta-analysis showed that compared with younger subjects, sarcopenia had a higher impact on people 79 years of age or older (*p* = 0.02) and was not only related to decreased muscle function (total OR 3.03, 95% CI: 1.80–5.12) but also related to higher mortality (overall odds ratio [OR] is 3.596, 95%) CI: 2.96–4.37) (Beaudart et al., 2017). Sarcopenia also causes an increase in the risk of falls and fractures, reduces quality of life, and increases disease. The mortality rate brings a heavy burden on the economy and health care system. Therefore, this disease should be taken seriously and treatment methods should be explored.

Bones and muscles are important parts of the movement system. The bones throughout the whole body are connected in different ways to form the skeleton, support body weight, protect internal organs, maintain body posture, and form the basic shape of the human body. They also provides wide attachment points for skeletal muscles (Avin et al., 2015). Muscle is the power device of the motion system. Muscles straddle one or more joints, contract, and pull the bones attached to them, producing a lever motion via the bone connection under control of the nervous system (Avin et al., 2015). In our understanding, the coupling of bone and muscle is mechanical. However, in recent years, the understanding of bone and muscle has been further improved. It seems that the two are not simply connected but have a deeper level of communication. The present review discusses in detail the deep-level connection between bones and muscles. As an endocrine organ, what kind of effect do secreted bone factors have on muscle? Is there a certain therapeutic significance for sarcopenia?

## What Is Sarcopenia?

In 1988, at a conference in Albuquerque, New Mexico, Rosenberg proposed sarcopenia as an age-related decrease in muscle mass (Rosenberg, 1997). With deepening of the understanding of sarcopenia, it was found that muscle loss includes not only a decrease in muscle mass but also a decrease in muscle strength (Mitchell et al., 2012). There are currently two main criteria for clinical diagnosis of sarcopenia: low muscle function (characterized by strength) and decreased muscle mass (Cruz-Jentoft et al., 2010, 2019; Chen et al., 2020).

Other syndromes, such as cachexia and starvation, will also show symptoms of muscle wasting, but sarcopenia is still different from these syndromes. In patients with primary sarcopenia, as age increases, for example, from the age of 20 to 80, muscle mass decreases by approximately 30%, and cross-sectional area decreases by approximately 20%, mainly due to the decrease in size and number of muscle fibers (Frontera et al., 2000). The cross-sectional area of muscle fibers is mainly occupied by type 2 muscle fibers. With age, type II fibers are preferentially lost, showing selective atrophy, while type I fibers are retained (Larsson et al., 1978). With age, motor units also decrease. Some scholars have confirmed that the number of motor units for people over 60 is approximately half that of people under 60 (Lexell et al., 1983; Brown et al., 1988). Due to insufficient protein and energy intake, starvation will lead to loss of body fat and non-fat substances, but through supplementation of protein and energy, the symptoms will be improved (da Silva et al., 2020). Cachexia is widely considered to be a disease state accompanied by severe weight loss caused by cancer and other chronic wasting diseases, and the main feature is muscle loss, with or without fat loss (Evans et al., 2008). In addition, cachexia is usually accompanied by inflammation, insulin resistance, and increased muscle protein breakdown (Durham et al., 2009; Morley et al., 2009). Therefore, most individuals with cachexia also present with sarcopenia, specifically, secondary sarcopenia, but most age-related primary sarcopenia is not considered cachexia. Sarcopenia is only one element of cachexia.

Currently, for sarcopenia, there are already some treatment measures; for example, some of the adverse effects of sarcopenia can be reduced by adaptive physical exercise (Anton et al., 2018; Liao et al., 2019; Nascimento et al., 2019; Billot et al., 2020). Evidence shows that the muscle mass and strength of patients with sarcopenia can be increased by participation in resistance exercise training (Csapo and Alegre, 2016; Vlietstra et al., 2018). Appropriate nutritional intervention can improve sarcopenia caused by nutritional deficiencies (such as a lack of protein or vitamin D) and improve muscle mass and strength (Bauer et al., 2015; Hickson, 2015). β-Hydroxy-β-methylbutyrate (HMB) has been shown to improve muscle mass and maintain muscle strength and function in elderly people with sarcopenia or weakness (Bear et al., 2019). With age, the endocrine factors that affect muscle protein synthesis will have an increasing impact, resulting in a decrease in the number of muscle fibers, muscle cross-sectional area, and skeletal muscle mass. Over the past decade, several studies have shown that the effect of bones on muscles exceeds the scope of machinery. In osteoblast/osteocyte-deficient connexin 43 (Cx43) mice, some defective muscle phenotypes and the cross-sectional area and grip strength of the extensor digitorum longus muscle were partially rescued via subcutaneous injection of the bone-specific factor carboxylated osteocalcin (Shen et al., 2015). The expression of the full propeptide (IGF-1Ea) of IGF-1 protects against age-related loss of muscle mass and strength (Ascenzi et al., 2019). Denosumab, a RANKL blocking antibody, prevents RANKL from binding to RANK, thereby reducing cytokines (such as TNF) that cause muscle wasting and apoptosis (Bonnet et al., 2019). Therefore, a better understanding of the molecular mechanism underlying the messenger action produced by bone tissues will help in future development of new treatments for sarcopenia (Table 1).

**TABLE 1:** Therapeutic directions for sarcopenia in the future.

Bone-derived cytokines	Therapeutic directions	References
FGF-23	Anti-FGF-23 neutralizing antibody	Aono et al. (2011)
RANKL	Denosumab (a RANKL blocking antibody)	Lefkowitz et al. (2012), Bonnet et al. (2019)
	Exogenous OPG	Bonnet et al. (2019)
	Knock out the muscle RANK gene	Dufresne et al. (2016a)
IGF-1	Resistance training reverses IGF-1 decline	Yoon et al. (2019)
	IGF-1 that simulates supramolecular nanofiber/hydrogel formed by Nap-FFGSSSR	Shang et al. (2020)
	The full propeptide IGF-1Ea and IGF-1Eb of IGF-1	Ascenzi et al. (2019)
	Losartan	Burks et al. (2011)
Osteocalcin	Exogenous ucOC	Baskin et al. (2015)
	The osteoblast/osteoblast Cx43 gene (Gja1)	Shen et al. (2015)

## Bone-Muscle Biochemical Crosstalk

Muscles and bones interact to maintain their structures and functions. Thus far, we have realized that muscles and bones can receive and secrete biochemical signals in a two-way manner, thereby affecting the metabolism of the two tissues and the entire body (Lombardi, 2019; Gomarasca et al., 2020). These signals are coordinated by a set of cytokines and growth-like factors: muscle factors secreted by muscle cells and bone factors secreted by bone cells, both of which can exert autocrine, paracrine and endocrine functions to regulate muscle and bone metabolism.

Diseases characterized by changes in muscle physiology affect bone function and structure, and vice versa. The effects of muscle on bone have been intensively studied. Skeletal muscle can be identified as an endocrine organ that produces secretory factors. Muscle-derived factors are called myokines and were first proposed by Pedersen and colleagues in 2010 (Pedersen, 2011). These molecules include myostatin, interleukin 6 (IL-6), interleukin 8 (IL-8), interleukin 15 (IL-15), leukemia inhibitory factor (LIF), brain-derived neurotrophic factor (BDNF), follistatin-like protein 1, fibroblast growth factor 21 (FGF-21), and irisin, which act in an autocrine, paracrine or endocrine manner. Many of these muscle factors can significantly affect bone repair and bone metabolism. For example, myostatin can inhibit skeletal muscle mass and development (Lee and Jun, 2019) and has a negative regulatory effect on bone mass (Kaji, 2016; Qin et al., 2017). Sclerostin is a secreted glycoprotein expressed by bone or cartilage cells that inhibits bone formation via the Wnt/β-catenin pathway (van Bezooijen et al., 2004). The latest research has found that skeletal muscle is a new source of sclerostin. Muscle-derived sclerostin works synergistically with bone-derived sclerostin to strengthen the negative regulatory mechanism of osteogenesis (Magarò et al., 2021). This further strengthens the team of myogenic factors and gives us a better understanding of muscles.

**FIGURE 1:**
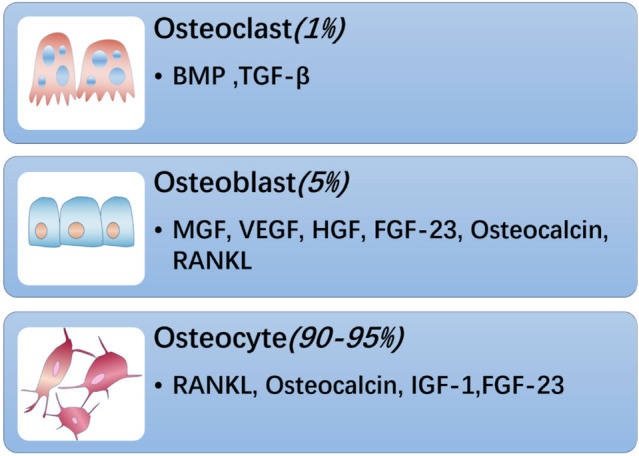
Bone is composed of osteoblasts, osteoclasts and osteocytes. Among them, osteoclasts account for about 1%, which can secrete BMP, TGF-β and other substances; osteoblasts account for 5%, secrete bone-derived factors such as MGF, VEGF, HGF, FGF-23, RANKL, and osteocalcin, and osteocytes account for the largest proportion, About 90–95%, can secrete FGF-23, RANKL, IGF-1, osteocalcin, etc.

**FIGURE 2:**
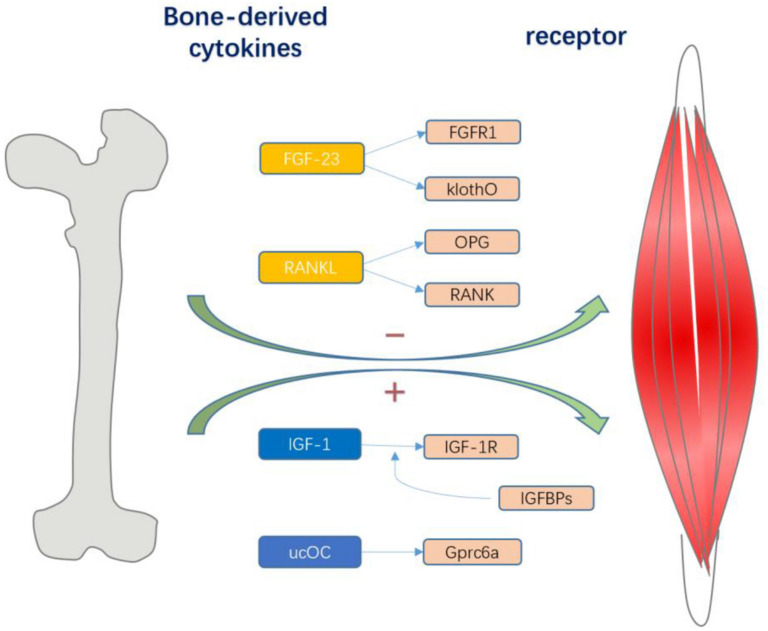
Various bone cells can secrete different bone-derived factors. Osteocalcin regulates exercise through Gprc6a signal transduction in muscle fibers, which also contributes to the nutrient absorption and catabolism of muscles during exercise; and IGF-1 acts on IGF-1R to activate downstream signaling pathways and determines its biological activity. IGFBPs sterically hindered the interaction of IGFs with the IGF-I receptor, and they were potent competitive inhibitors. Both thus have a positive effect on skeletal muscle. And FGF23 and RANKL can have a negative effect on skeletal muscle. The combination of FGF23 with FGFR1 and the co-receptor Klotho stimulates downstream effects, and the RANKL/RANK interaction activates NF-kB, and OPG, as a soluble receptor of RANKL, prevents it from binding to RANK.

There are three cellular components in adult bone. Osteoblasts and osteoclasts account for approximately 5 and 1% of the cells, respectively, whereas the remaining 90–95% are osteocytes (Figure 1). Osteoblasts are specialized mesenchymal cells that synthesize bone matrix and coordinate bone mineralization, which play a key role in regulating bone metabolism (Boskey, 1996; Neve et al., 2011). Transforming growth factor β (TGF-β), bone morphogenetic protein (BMP), osteocalcin and insulin-like growth factor (IGF1) are expressed in the bone matrix by osteoblasts and released by osteoclasts during the absorption process (Linkhart et al., 1996). However, bone cells are neglected and are considered to be true secretory osteocytes. It was first proposed that osteocytes are endocrine cells in 2006, and the first secreted factor identified was fibroblast growth factor 23 (FGF23), which is highly elevated in osteocytes of patients with hypophosphatemic rickets (Feng et al., 2006; Liu et al., 2006). After more than 10 years of exploration and research, bone has gradually been recognized as an endocrine organ that can secrete a variety of osteokines, such as sclerostin, PGE2, Dickkopf-1 (Dkk1), stromal extracellular phosphoglycoprotein (MEPE), and osteoprotegerin (OPG), and small molecule adenosine triphosphate (ATP) and nitric oxide (NO), which have important effects on bones (Dallas et al., 2013; Buenzli and Sims, 2015; Han et al., 2018). In addition, “osteokines,” such as osteocalcin, nuclear factor-kappa B receptor activator ligand (RANKL), insulin-like growth factor (IGF-1), and fibroblast growth factor 23 (FGF-23) play an important role in the quality and function of muscles (Figure 2). Notably, lipocalin 2 (LCN2), a fat factor that was previously considered to be associated with obesity (Soukas et al., 2000; Lin et al., 2001; Yan et al., 2007) was recently found to exhibit an expression level in osteoblasts that is at least 10 times higher than that in white fat. LCN2 crosses the blood–brain barrier and interacts with melanocortin 4 receptor (MC4R) in the hypothalamus to inhibit appetite after binding (Mosialou et al., 2017) which further supports the idea that bone can play a corresponding role as an endocrine organ.

Although our understanding of the biochemical communication between bones and muscles has gradually increased and sarcopenia has become increasingly common in elderly patients, we currently have no specific measures or methods for treatment and prevention of sarcopenia; thus, it is necessary to explore whether osteokines can be used to treat and prevent this disease. Many bone-derived factors have been discovered, but here, we mainly focus on four bone factors that have a regulatory effect on muscles, namely, osteocalcin, IGF-1, RANKL and FGF-23, and seek to understand their possible therapeutic targets in sarcopenia.

### Osteocalcin and Its Effect on Muscles

Osteocalcin is the most abundant non-collagen protein in the bone matrix and is mainly produced by mature osteoblasts (Hauschka et al., 1989). Osteocalcin has a high affinity for hydroxyapatite and is responsible for its storage in bone (Hauschka et al., 1989). However, due to its low pH and decarboxylation, it can be released into the circulation system. Its concentration level in the circulation system is controlled by the circadian rhythm. Human osteocalcin levels are very low in the morning and begin to rise in the afternoon, reaching a peak in the evening. The main form of osteocalcin in serum is incompletely carboxylated (OC with insufficient carboxyl groups) (ucOC) (Ferron et al., 2010), which is an uncarboxylated form and is related to glucose homeostasis in mice. The circulating level of ucOC is increased after exercise (Fernández-Real et al., 2009; Lin et al., 2012; Pernambuco et al., 2013; Kim et al., 2015; Ahn and Kim, 2016), and the increase in ucOC is related to the decrease in serum glucose levels after exercise (Lin et al., 2012). Since skeletal muscle is a major part of glucose processing (Holloszy, 2005; Funai et al., 2009), ucOC may be related to skeletal muscle function in both direct and indirect ways.

Osteocalcin has many functions in mice, such as regulating glucose metabolism, energy metabolism, fertility and ectopic calcification. Recent studies have shown that osteocalcin also has an effect on muscle (Levinger et al., 2014), thereby affecting the entire body’s physiology (Plant and Lynch, 2002; Karsenty and Ferron, 2012). Gprc6a may be the receptor of ucOCN (Mera et al., 2016a; Pi et al., 2016; Liu et al., 2017). Karsenty et al. analyzed mutant mouse strains lacking osteocalcin and/or its receptor Gprc6a in a cell-specific manner. Their results showed that muscle function during exercise requires Gprc6a calcium signaling in the bone and in muscle fibers. Exogenous osteocalcin cannot correct the poor exercise capacity in Gprc6aMck-/- mice and Ocn ± mice. Gprc6aMck ± mice showed the same decline in exercise capacity as Ocn-/- mice. In Gprc6aMck-/- mice, osteocalcin was identified as the main ligand of Gprc6a (responsible for the muscle function regulation and exercise adaptation activity of Gprc6a). Through the signal transduction of Gprc6a in muscle fibers, osteocalcin is a system modulator allowing adult mice to adapt to exercise (Mera et al., 2016a). Therefore, the osteocalcin signal in muscle fibers is not only necessary for adapting to exercise but also aids in muscle nutrient absorption and catabolism during exercise. First, osteocalcin signaling in muscle fibers contributes to the breakdown of glycogen, which is the main source of glucose for muscle contraction during exercise. Second, it promotes transport of the glucose transporter GLUT4 to the plasma membrane, thereby enhancing glucose uptake and glycolysis. Third, osteocalcin signal transduction in muscle fibers can increase the uptake and catabolism of fatty acids (FAs). Therefore, osteocalcin signaling in muscle fibers provides the necessary carbon atoms to promote the Tricarboxylic Acid Cycle (TCA) cycle and thereby generate ATP for increased muscle function (Mera et al., 2016a).

The maintenance of muscle mass depends on a balance between anabolic (protein synthesis) and catabolic (muscle breakdown) events, which together determine the level of muscle protein. Karsenty’s research team observed that osteocalcin signaling in the muscle fibers of aged mice promoted protein synthesis (Mera et al., 2016b). Other muscle anabolic hormones can activate the PI3K/Akt/mTOR pathway, thereby stimulating protein synthesis and muscle hypertrophy (Rommel et al., 2001). This is consistent with their previous results showing that osteocalcin plays a pivotal role in stimulating Akt phosphorylation in muscle during exercise (Mera et al., 2016a). Similarly, Suifeng Liu’s team discovered that ucOC promoted myoblast proliferation through the PI3K/Akt/p38 MAPK pathway. Gprc6a-Erk1/2 signaling promotes myogenic differentiation (Liu et al., 2017). Xuzhu Lin et al. found that in the extensor digitorum longus (EDL) muscle, osteocalcin enhances p-AktSer473 by increasing the total Akt expression level; in the soleus muscle, osteocalcin mimics the phosphorylation of PKC (protein kinase C), which may lead to increased activation of Akt and AS160 under insulin stimulation (Lin et al., 2017). In other organs of the human body, including pancreatic islets, testicular stromal cells and the brain, osteocalcin combines with its receptor Gprc6a to activate downstream signaling pathways, which is beneficial to protein synthesis and/or cell proliferation (Lee et al., 2007; Wei et al., 2014).

After a long period of regular exercise regardless of any form, body fat is reduced, insulin resistance is improved, and serum total osteocalcin and decarboxylated osteocalcin are significantly increased (Fernández-Real et al., 2009; Lin et al., 2012; Pernambuco et al., 2013; Kim et al., 2015; Ahn and Kim, 2016) but an increase in osteocalcin can also be observed after 5 min of exercise. The transient increase in the serum osteocalcin level does not appear to be an activity mediated by osteoblasts (Lin et al., 2012). This may be due to the influence of mechanical load. Studies have found interesting things; for example, with age, mice lacking insulin receptors (IRs) in osteoblasts showed significant peripheral obesity and insulin resistance compared with control mice in the same litter (Fulzele et al., 2010). At the same time, this phenotype was accompanied by a decrease in the lack of carboxylation of osteocalcin in the circulation. These conditions can also be observed in osteocalcin-deficient mice. Intermittent injection of exogenous osteocalcin promotes an increase in β-cell mass and insulin secretion, thereby significantly improving abnormal metabolism (Fulzele et al., 2010; Ferron et al., 2012; Huang et al., 2017). In mice taking osteocalcin daily, liver steatosis caused by a high-fat diet was completely cured, which also confirmed that daily injection of osteocalcin can improve the body’s ability to process glucose and prevent the development of type 2 diabetes.

Karsenty et al. found that IL-6 is a target gene for osteocalcin in muscle. Circulating levels of this muscle factor increase during exercise and enhance exercise capacity. In turn, IL-6 facilitates adaptation to exercise, in part by signaling in the bone to increase osteoclast differentiation and the production of bioactive osteocalcin (Mera et al., 2016a). Mice lacking IL-6 receptors in osteoblasts showed severe motor deficits, similar to mice lacking myogenic IL-6 (mIL-6). This deficiency can be caused by osteocalcin rather than IL-6. Recently, some researchers have used ^3^H-2-deoxyglucose (^3^H-2-DG) uptake to measure glucose uptake and found that the uptake of ^3^H-2-DG was decreased in oxidized muscle in both IL6_Hsa_^–/–^ and IL6r_Osb_^–/–^ mice compared with their respective control litters. Moreover, the expression of Pgma1, which is necessary for glycolysis in the oxidative muscles of IL6_Hsa_^–/–^ mice, decreased after exercise, and a similar reduction in glucose uptake was observed in Ocn^±^ IL6_Hsa_^±^ and Ocn^±^ IL6r_Osb_^±^ mice. All these results indicate that osteocalcin-mediated mIL-6 has a positive effect on muscle fiber glucose uptake during exercise. Osteocalcin was injected into IL6_Hsa_^–/–^, IL6r_Osb_^–/–^, Ocn^±^ IL6_Hsa_^±^, or Ocn^±^ IL6r_Osb_^±^ mice, and glucose uptake in the muscles of all the mutant mouse strains was found to be normalized. In addition, during exercise, mIL-6 promotes nutrient absorption and catabolism in muscle fibers in an osteocalcin-dependent manner. Compared with control litter mice before and after exercise, IL6_Hsa_^–/–^ and IL6r_Osb_^–/–^ mice have normal or increased circulating levels of non-esterified fatty acids (NEFAs) and triglycerides. Similarly, comparing IL6_Hsa_^–/–^, IL6r_Osb_^–/–^, Ocn^±^ IL6_Hsa_^±^, or Ocn^±^ IL6r_Osb_^±^ mice with their respective control groups after exercise, it was found that the expression levels of FATP1, which promotes the uptake of long-chain FAs into cells, and of CPT1B, which facilitates FA transport across the mitochondrial membrane (Stahl et al., 2001), are significantly reduced in muscles (Chowdhury et al., 2020). Between humans and rodents, the crosstalk between osteocalcin and IL-6 is conserved, indicating that the musculoskeletal-muscle endocrine axis is an essential part of enhancing muscle function in humans and rodents.

#### The Future Direction of Osteocalcin in Treatment of Sarcopenia

Osteocalcin can actively regulate exercise capacity, and its level drops sharply during aging. This fact suggests that osteocalcin may regulate muscle mass. By analyzing mutant mice lacking osteocalcin or its receptor Gprc6a, it was found that this regulatory effect of troponin signaling in muscle fibers could not be detected in young mice, indicating the presence of other potential mechanisms besides osteocalcin signaling. Osteocalcin signaling works to maintain the muscle mass of young mice but also improves the exercise capacity of 3-month-old mice. As age increases, the circulating osteocalcin level decreases sharply. Thus, whether osteocalcin is administered acutely or chronically, the exercise ability of 9-, 12-, or even 15-month-old mice is restored to that of 3-month-old mice (Mera et al., 2016a). This result indicates that osteocalcin signal transduction in muscle fibers is a novel and powerful means to combat age-related declines in muscle function. However, due to the increasing number of molecules that affect muscle function (Baskin et al., 2015) it is important to determine whether osteocalcin works in synergy with some of these molecules to promote adaptation to exercise. Specific excision of the mouse osteoblast/osteocyte Cx43 gene (Gja1) affects the development of skeletal muscle, resulting in a decrease in rapid muscle weight, grip strength, and maximum absolute and specific tonicity, as well as differences in osteocalcin activity and insufficient carboxylation. The construct promoted the formation of myotubes in C2C12 myoblast cultures, and injection of osteocalcin into the mice rescued the cross-sectional area and grip strength of the extensor digitorum longus muscle (Shen et al., 2015). Hypocarboxylated osteocalcin was employed in mice receiving short-term glucocorticoids (GCs). It was found that ucOC treatment can improve the muscle insulin sensitivity of mice receiving short-term cortisol (CS) administration. The underlying mechanism of this ucOC effect involves enhancing the activation and abundance of key proteins in the distal insulin and ucOC signaling pathways in a distal muscle-specific manner (Lin et al., 2019). In animal and preclinical studies, ucOC has been found to have a promising role in improving muscle metabolism and function, but the role of ucOC in humans and its relationship with muscle function and metabolism are still unknown. Although many functions of osteocalcin are not very relevant, its ability to improve muscle function and promote an acute response to stress during exercise indicates that this unique bone-derived hormone presents a survival advantage. A deeper understanding of bone-derived hormones is still required. From a broader and longer-term perspective, this may slow or even reverse the onset of age-related diseases.

### IGF-1 and Its Effect on Muscles

Insulin-like growth factor-1 (IGF-1) is a 70-amino acid single-chain peptide with a molecular weight of 7.6 kDa. IGF-1 contains three disulfide bonds between amino acids 6 and 48, 18 and 61, and 47 and 52, which form a tertiary structure (Sjögren et al., 1999; Yakar et al., 1999) and are essential for optimal binding to IGF-1R. IGF-1 commonly serves as an endocrine hormone that is mainly secreted by the liver and transported to target tissues. It is also produced by the local action of extrahepatic tissue in a paracrine manner; for example, bone tissue produces IGF-1 to act on skeletal muscle (Sheng et al., 2013). IGF-1 is used as a marker of medical conditions and diseases, such as acromegaly, breast cancer (Peyrat et al., 1993; Hankinson et al., 1998; Tanimoto et al., 2008; Murphy et al., 2020), prostate cancer (Stattin et al., 2004; Rowlands et al., 2009), type 1 and type 2 diabetes (T2DM) (Mastrandrea et al., 2008; Cubbon et al., 2016), heart disease (Laughlin et al., 2004), non-alcoholic fatty liver disease (NAFLD) (Brea et al., 2005) and sepsis (de Groof et al., 2002). Here, we discuss its effects on skeletal muscle.

The growth hormone/insulin-like growth factor (IGF) axis is an important determinant of muscle mass and function (Drey, 2011). IGF-1 is both hyperplastic and hypertrophic in skeletal muscle. The hyperplastic effect results in the proliferation of muscle satellite cells, which donate their nuclei to multinucleated myofibers. The hypertrophic effect results in increased synthesis of contractile proteins by existing myonuclei. Local IGF-1 is primarily secreted by bone cells, which has little effect on the level of IGF-1 in blood circulation and is mainly involved in bone transformation (Sheng et al., 2013). Bone cell-specific conditional IGF-1 gene knockout mice manifested decreased muscle mass, and the expression of IGF-1 mRNA in muscle was decreased by 59%, suggesting that local IGF-1 may be involved in regulating muscle metabolism. IGF-1Ec, an isoform of IGF-1, also called mechanical growth factor (MGF) (Matheny et al., 2010) is very sensitive to mechanical stimulation. It is significantly upregulated after exercise training and skeletal muscle injury (Matheny et al., 2010). MGF can activate muscle satellite cells, promote the proliferation of myoblasts, maintain the quality of local skeletal muscle, and promote the repair of damaged tissues.

IGF-1 binds to IGF-1 receptors (IGF-1Rs) on the muscle fiber membrane to initiate a signal for muscle protein synthesis. If the number of IGF-1 receptors decreases and the circulating IGF-1 hormone levels remain unchanged, downstream events will stop, thereby affecting protein synthesis. The activity of IGF-1 is tightly controlled by a family of plasmatic transportation proteins called insulin-like growth factor–binding proteins (IGFBPs) (Frystyk, 2004; Jogie-Brahim et al., 2009). The IGFBP family may help improve function and regulate the level of diversity, thereby promoting the fine-tuning of IGF biological activity and signal transduction (Allard and Duan, 2018). The IGFBP family consists of six IGFBPs, IGFBP1 to IGFBP6, and other proteins with low binding affinity to IGFs are called IGFBP7, IGFBP8, and IGFBP9 (Ding and Wu, 2018). Insulin-like growth factor binding protein-1 (IGFBP-1) can be a determinant of IGF-1 activity. In previous studies, it was found that the level of IGFBP-1 was negatively correlated with that of free IGF-1 (Frystyk, 2004). In a cross-sectional study of the relationship between serum IGFBP-1 and muscle mass in elderly women between 55 and 85 years old, researchers such as Alicja Wolk observed that IGFBP-1 is positively correlated with low relative muscle mass (Stilling et al., 2017). Compared with IGFBP-1, IGFBP-2 and IGFBP-3 not only participate in the pathological process of most human diseases, such as prostate cancer, lung cancer and other malignant diseases, blocking them seems to be an effective way to inhibit tumor growth and metastasis and can also improve metabolism, such as inhibiting fat production and enhancing insulin sensitivity. The IGFBP family has a certain ability to regulate IGF-1. Whether there are other members in addition to IGFBP-1 that affect muscles is worth our continued exploration.

Regulating protein synthesis in skeletal muscle and promoting body growth is one of the most important functions of IGF-1. Through *in vitro* experiments, Rommel, C. and other researchers found that the PI3K/Akt/mTOR and PI3K/Akt/GSK3 pathways mediate IGF-1-induced skeletal muscle hypertrophy. The main processes are as follows: After IGF-1 binds to the IGF-1 receptor (IGF-1R), it phosphorylates the intracellular adaptor protein insulin receptor substrate-1 (IRS-1), which recruits and phosphorylates phosphoinositide 3-kinase (PI3K); then, protein kinase B (Akt) is phosphorylated. Akt promotes hypertrophy by activating downstream signaling pathways involved in protein synthesis: one is to promote protein synthesis through the downstream pathway of the mammalian target of rapamycin (mTOR), and the other pathway activates phosphorylation to inhibit glycogen synthase kinase 3 (GSK3), thereby activating Eif2b and transcriptional activator β-catenin protein (Desbois-Mouthon et al., 2001; Rommel et al., 2001; Armstrong and Esser, 2005). In contrast, in addition to verifying that calcineurin does not mediate the hypertrophy induced by IGF-1, this study also demonstrated that IGF-1 unexpectedly antagonizes the calcineurin signal through Akt during myotube hypertrophy. At the same time, the results of *in vivo* studies have confirmed this. Researchers have found that activation of mTOR through PI3K/Akt may be an important regulator of muscle fiber growth in the body (Bodine et al., 2001). Activated Akt can not only increase the fiber size in normal muscles but can also maintain the size of muscle fibers in atrophic muscles. This shows that IGF-1 is a key intracellular signaling protein that promotes skeletal muscle growth and performance. In contrast, IGF-1-mediated phosphorylation of Forkhead box O (FoxO) inactivates this pathway, and dephosphorylated FoxO is translocated into the nucleus, where it induces the transcription of atrogin-1, MuRF-1 and other genes that cause muscle protein breakdown (Sandri et al., 2004; Stitt et al., 2004). Therefore, low levels of IGF-1 are related to a decrease in PKB/AKT and mTOR and an increase in the transcription factor FoxO. We can use IGF-I to reduce the effect of the cell cycle inhibitor p27Kip1 through the PI3K/Akt pathway, thereby promoting the proliferation of satellite cells and regeneration of aging muscles (Chakravarthy et al., 2001). In a recent study, Sullivan et al. (2020) found that both men and women with obesity had a lower IGF-1 level in skeletal muscle at rest and after acute resistance exercise compared with lean counterparts. However, lower IGF-1 expression was not related to lower downstream signaling through Akt and mTOR. A lower resting IGF-1 mRNA level was found to be correlated with a greater miR-206 level, indicating possible epigenetic regulation of muscle IGF-1 expression (Sullivan et al., 2020). In the disuse state (not with age), the IGF-1/PI3K/Akt signaling pathway is weakened. Meanwhile, the serum IGF-1 and IGFBP-3 concentrations can be detected in a low state (Silva-Couto Mde et al., 2014; Timmer et al., 2018). Therefore, the above pathway may alleviate skeletal muscle atrophy in some cases.

Adult non-growing skeletal muscle is difficult to hypertrophize in response to elevated IGF-1. In contrast, during muscle growth, the protein content of muscle fibers is increased through activation of signaling downstream of the IGF-1 receptor (Akt, phosphorylation of p70S6K) (Shavlakadze et al., 2010). In other words, stimulation of IGF-1 may induce skeletal muscle hypertrophy. Betaine supplementation may enhance the skeletal muscle differentiation of mouse myoblasts by activating IGF-1 signaling *in vitro* (Senesi et al., 2013). One of the factors potentially shared between muscle and bone is IGF-1. Thus, we must continue in-depth exploration of IGF-1.

#### The Future Direction of IGF-1 in Treatment of Sarcopenia

The circulating IGF-1 level decreases during the aging process (Bucci et al., 2013). In a cross-sectional study of elderly people in the community in Singapore, reduced IGF-1 levels coexisted with reduced vulnerability and muscle mass. In contrast, it is different in males, indicating that IGF-1-dependent anabolic pathways may be dominant in women (Chew et al., 2019). Serum IGF-1 was significantly lower among female sarcopenic subjects, with a demonstrable trend for a protective effect against sarcopenia in multiple regression models, such that each 1 ng/ml increase in IGF-1 was associated with a 1% decline in the odds of sarcopenia in women (*p* = 0.095) (Tay et al., 2015). In another cross-sectional study, it was found that women over 60 years of age with hip fractures had a high incidence of sarcopenia, and low serum IGF-1 and insulin-like growth factor binding protein-3 were detected in these women (Yee et al., 2020). This shows that IGF-1 may be used as a potential biomarker of sarcopenia. Age-related downregulation of the skeletal muscle IGF-1 system may be reversed to some extent with progressive resistance training (Urso et al., 2005; Chen et al., 2017; Yoon et al., 2019). A recent study confirmed that resistance interval training (RIT) and resistance aerobic exercise (RAE) can effectively improve physical health and sleep quality by increasing the area of skeletal muscle and IGF-1 in elderly women (Yoon et al., 2019). A supramolecular nanofiber/hydrogel formed by Nap-FFGSSSR mimics IGF-1 and can increase the phosphorylation of Akt by activating the insulin-mediated signaling pathway, which effectively promotes the proliferation of myoblasts, significantly reduces the apoptosis of myoblasts induced by dexamethasone, helps the myoblasts to differentiate into myotubes, and prevents the fibrosis of muscle tissue and the deposition of collagen;(Shang et al., 2020) these results show a prominent effect of IGF-1 in the treatment of sarcopenia. The expression of the full propeptide IGF-1Ea of IGF-1 promotes a significant hypertrophic phenotype in young mice and maintains this phenotype in older mice. However, inspections of aging transgenic mice showed that local expression of the IGF-1Ea or IGF-1Eb transgene has a protective effect on age-related loss of muscle mass and strength (Ascenzi et al., 2019). Losartan can counteract disuse atrophy in old mice with fixed hind limbs and prevent loss of muscle mass (Burks et al., 2011). This protective mechanism is due to increased activation of the IGF-1/Akt/mammalian Ray mTOR pathway, which blocks AT1 (vascular tension) (Prime type) receptors can improve muscle remodeling and prevent disuse atrophy and may prove to have clinical benefits against injury-related muscle remodeling and provide prevention of disuse atrophy for people with secondary sarcopenia protection.

### RANKL and Its Effect on Muscles

Receptor activator of nuclear factor kappa-B ligand (RANKL), also known as tumor necrosis factor ligand superfamily member 11 (TNFSF11), TNF-related activation-induced cytokine (TRANCE) and bone protein-ligand (OPGL), was first identified as a product of immune cells. Moreover, it has also been considered an important indicator of osteoclast differentiation (a membrane-bound factor expressed by osteoclasts to form supporting cells, such as osteoblasts and osteocytes) (Yasuda et al., 1998; Nakashima et al., 2011; Xiong et al., 2011).

RANKL and its RANK are an upstream signaling pathway of nuclear factor-κB (NF-κB). NF-κB is a key transcription factor that induces many proinflammatory genes, and its expression is upregulated in muscular dystrophy. Specific inhibition of NF-κB activity can reduce damage, inflammation and fibrosis of dystrophic muscle (Kumar and Boriek, 2003; Mourkioti et al., 2006; Acharyya et al., 2007; Yin et al., 2017). In bone, the RANKL/RANK interaction activates NF-κB, induces the formation of multinucleated mature osteoclasts, and causes bone resorption (Boyce et al., 2015). Increased levels of RANKL in menopausal women play a major role in the occurrence of osteoporosis, since the molecule can activate cell differentiation by binding to its receptor (RANK) and improves the activity and survival rate of osteoclasts (Eghbali-Fatourechi et al., 2003; Lacey et al., 2012). Osteoprotegerin (OPG), a soluble receptor of RANKL, prevents it from binding to RANK, thereby inhibiting osteoclast production. RANK in muscle is a key regulator of Ca2 + storage, SERCA activity and the rapid contraction of skeletal muscle. The RANKL-RANK interaction regulates Ca2 + storage and thus has an effect on muscle performance (Dufresne et al., 2016b). It has been found that genetic deletion of dystrophic muscle RANK and short-term selective inhibition of RANKL can significantly improve the muscle strength and integrity of young dystrophic MDX mice, such as in the dystrophic extensor muscle (EDL) and soleus muscle (Sol) (Bonnet et al., 2019; Hamoudi et al., 2019). Among them, anti-RANKL treatment preserves muscle integrity, reduces the damage and fiber of the dystrophic muscle, and can increase the mechanical properties of the bone in dystrophic mice (Hamoudi et al., 2019). RANK and/or RANKL can also be expressed in skeletal muscle, and this activation mainly inhibits myogenic differentiation, resulting in skeletal muscle dysfunction (Langen et al., 2001; Lee and Goldberg, 2015). In turn, in MDX mice (Duchenne muscular dystrophy mouse model), exogenous OPG has been shown to reduce inflammation and restore skeletal muscle function (Dufresne et al., 2015, 2016b). Using full-length OPG-Fc to improve dystrophic muscle function has a certain superiority over use of truncated OPG-Fc, anti-RANKL antibody, or anti-TRAIL antibody and muscle RANK loss (Dufresne et al., 2018).

#### The Future Direction of RANKL in Treatment of Sarcopenia

Denosumab (Dmab), a RANKL blocking antibody that mimics the effects of OPG, has been demonstrated to reduce the risk of fractures and is widely used in the treatment of osteoporosis (McCloskey et al., 2012). A recent study found that there was no significant difference in bone or muscle parameters in postmenopausal women with osteoporosis treated with denosumab or bisphosphonate. However, only denosumab improved muscle strength. A higher level of RANKL expression not only induced bone loss but also concomitantly impaired muscle structure, strength, and glucose uptake *in vivo* (Bonnet et al., 2019). Moreover, RANKL inhibitors (such as OPG-Fc and Dmab) corrected these abnormalities in both RANKL mice and Pparb-/-osteo lipoprotein-deficient mice, which indicates that the RANKL-RANK system is ultimately related to muscle weakness (related to the development of the system, instead of the trigger mechanism) (Bonnet et al., 2019). Stanley S et al. used the fully human monoclonal antibody denosumab in a patient with facial scapular humeral muscular dystrophy and achieved good therapeutic effects (Lefkowitz et al., 2012). This may be explained by the function of denosumab, which prevents binding of the RANK receptor by RANKL, with a resultant reduction in cytokines (e.g., TNF can cause muscle wasting and cellular apoptosis). Prior to this, some researchers confirmed that conditional knockout of RANK in muscle could prevent denervation-induced muscle weakness (Dufresne et al., 2016a). Taken together, the RANK/RANKL/OPG system may play an important role in muscle metabolism and the development of sarcopenia.

### FGF-23 and Its Effect on Muscles

Fibroblast growth factor 23 (FGF-23) is the first hormone-like osteokine found to be secreted by bone cells (Liu et al., 2006). FGF23 gene mutation is the cause of autosomal dominant hypophosphatemic rickets (ADHRs) (Econs et al., 1998). FGF23 and parathyroid hormone (PTH) can jointly regulate phosphate metabolism (Quarles, 2012). FGF-23 downregulates the expression of sodium/phosphorus co-transporter, which is responsible for the absorption and reabsorption of phosphate and acts on the proximal and distal tubules of the kidney to inhibit phosphate reabsorption (Gattineni et al., 2009). In addition, FGF23 can also inhibit the production of 1,25(OH)_2_ vitamin D3 by inhibiting 1a-hydroxylase (Nabeshima, 2008; Wolf, 2010) which can also lead to a phosphate waste effect, consequently resulting in poor bone mineralization under pathological conditions (children suffer from rickets, and adults suffer from osteomalacia). To regulate the reabsorption of phosphate, FGF23 binds to a complex of FGFR1 and the co-receptor Klotho to stimulate downstream effects (Kuro-o et al., 1997; Urakawa et al., 2006; Avin et al., 2018). Notably, FGF-23 and Klotho knockout mice exhibit the same premature aging phenotype, including vascular calcification (Desjardins et al., 2012), cardiac hypertrophy (Faul et al., 2011; Kuga et al., 2020), metabolic bone disease, and cognitive impairment (Shimada et al., 2004). A cross-sectional study of 2977 elderly people in the community showed that FGF23 levels were higher in older people, which was an independent risk factor for debilitating and pre debilitating states. This result suggests that FGF23 may have a certain negative biological effect (Beben et al., 2016). Skeletal muscle mesenchymal stem cells (MSCs) can not only promote the differentiation of co-cultured satellite cells into muscle-like cells (Joe et al., 2010) but also regulate the maintenance of muscle fibers (Roberts et al., 2013). Therefore, the interaction between MSCs from skeletal muscle and satellite cells may play an important role in skeletal muscle regeneration and homeostasis. Chisato Sato et al. conducted a study on the effects of FGF-23 on isolated human MSCs *in vitro*. They found that FGF-23 promoted the p53/p21 pathway to induce premature senescence of human skeletal muscle mesenchymal stem cells in a Klotho-independent manner, which supports its inhibitory effect (Sato et al., 2016). Studies have found that the FGF-23 concentration in hemodialysis patients is positively correlated with muscle mass index. Its effect on muscle is independent of s-Klotho, and it directly binds to FGF receptors in skeletal muscle (Fukasawa et al., 2014). Li et al. (2016) treated C57BL/6J mice with 100 mg/(kg⋅d) exogenous recombinant FGF23 twice a day for 3 consecutive days. They found that the exercise endurance of the mice improved. It was speculated that increased reactive oxygen species (ROS) expression and enhanced mitochondrial function might account for this finding.

#### The Future Direction of FGF-23 in Treatment of Sarcopenia

FGF-23 was the first endocrine factor found in bone; its level is higher in older people, and it has a certain negative effect on the body (Liu et al., 2006; Beben et al., 2016). Anti-FGF-23 neutralizing antibody can increase the blood phosphorus and 1,25(OH)_2_D levels in hypophosphatemia (Hyp) young mice and improve humerus and X-linked hypophosphatemic rickets/osteomalacia (XLH) in young Hyp mice (Aono et al., 2009). In addition, FGF-23Ab also increases muscle strength and spontaneous exercise frequency in adult Hyp mice (Aono et al., 2011). It is worth considering whether FGF23 has a direct effect on skeletal muscle and what kind of regulation it exerts. Does FGF-23 play a major role in aging muscle? Can these findings in mice also be applied to humans? Can inhibition of excessive FGF-23 activity help to improve biochemical, morphological and histological alterations in the muscle of patients with FGF-23-related hypophosphatemia? Does it improve the symptoms of muscle weakness and the quality of life? The answers to these questions remain unclear. Therefore, further research and deeper discussion are still needed to address the above issues.

## Conclusion

During the aging process, loss of muscle mass is partially attributed to a gradual decrease in the cross-sectional area of muscle fibers. Several mechanisms have been proposed to account for this phenomenon, including decreased circulating levels of anabolic hormones and growth factors, internal changes in age-related muscle properties, and an age-related decrease in physical activity. Research on the underlying mechanisms of age-related muscle loss aims to identify targets for drug discovery and to develop novel and effective methods to combat muscle loss. In this review, we comprehensively summarized the latest research progress on the effects of the bone-derived cytokines FGF-23, IGF-1, RANKL, and osteocalcin on muscle and the prospects for treatment of sarcopenia. Understanding the mechanical, cellular and molecular mechanisms underlying the biochemical communication between bone and muscle is of great significance for discovering potential novel therapies for age-related disorders. In the future, bone-derived factors might be considered in the treatment of sarcopenia and other muscle disorders.

## Author Contributions

WL and WfX collected the data, decided on the content, and wrote the manuscript. YZ and YL conceptualized this review, and revised the draft. WqX made contributions to the manuscript revision process and language revision process. XF and LP made the figures. HJ and YY designed the table. All authors consent to the final vision of the manuscript and are willing to take responsibility for the content of all works provided.

## Conflict of Interest

The authors declare that the research was conducted in the absence of any commercial or financial relationships that could be construed as a potential conflict of interest.

## Publisher’s Note

All claims expressed in this article are solely those of the authors and do not necessarily represent those of their affiliated organizations, or those of the publisher, the editors and the reviewers. Any product that may be evaluated in this article, or claim that may be made by its manufacturer, is not guaranteed or endorsed by the publisher.

## Abbreviations

FGF-23: fibroblast growth factor 23
IGF-1: insulin-like growth factor
RANKL: receptor activator of nuclear factor kappa-B ligand
OR: odds ratio
CI: confidence interval
HMB: β hydroxy β methyl butyrate
Cx43: connexin 43
IL-8: interleukin 8
IL-15: interleukin 15
LIF: leukemia inhibitory factor
BDNF: brain-derived neurotrophic factor
FGF-21: fibroblast growth factor 21
TGF-β: transforming growth factor β
BMP: bone morphogenetic protein
PGE2: Prostaglandin E2
Dkk1: Dickkopf-1
MEPE: stromal extracellular phosphoglycoprotein
OPG: osteoprotegerin
LCN2: lipocalin 2
MC4R: melanocortin 4 receptor
OC: osteocalcin
UcOC: uncarboxylated osteocalcin
FAs: fatty acids
TCA: tricarboxylic acid cycle
ATP: adenosine triphosphate
EDL: extensor digitorum longus
PKC: protein kinase C
IR: insulin receptors
mIL-6: myogenic IL-6
^3^H-2-DG: ^3^H-2-deoxyglucose
IGF-1R: insulin-like growth factor-1 receptor
MGF: mechanical growth factor
IGFBPs: insulin-like growth factor–binding proteins
IRS-1: insulin receptor substrate-1
Akt: protein kinase B
mTOR: mammalian target of rapamycin
GSK3: glycogen synthase kinase 3
FoxO: Forkhead box O
TNFSF11: tumor necrosis factor ligand superfamily member 11
TRANCE: TNF-related activation-induced cytokine
NF-κB: Nuclear factor-κB
MSCs: mesenchymal stem cells
NF-κB: Nuclear factor-κB
OPG: osteoprotegerin
Sol: soleus muscles
ADHR: autosomal dominant hypophosphatemic rickets
PTH: parathyroid hormone
MSCs: mesenchymal stem cells
ROS: reactive oxygen species.
